# *Caenorhabditis elegans* BRICHOS Domain–Containing Protein C09F5.1 Maintains Thermotolerance and Decreases Cytotoxicity of Aβ_42_ by Activating the UPR

**DOI:** 10.3390/genes9030160

**Published:** 2018-03-13

**Authors:** Myungchul Song, Kyunghee Song, Sunghee Kim, Jinyoung Lee, Sueyun Hwang, Chingtack Han

**Affiliations:** 1 Department of Life Science, Sogang University, Seoul 04107, Korea; gomsemarii@sogang.ac.kr (M.S.); kittensong@lgcare.com (K.S.); kshkwy@naver.com (S.K.); jinylee@amorepacific.com (J.L.); 2 LG Household & Health Care, Daejeon 34114, Korea; 3 Department of Medicine, Biomedical Research Institute, Seoul National University Hospital, Seoul 03080, Korea; 4 Amorepacific R&D Center, Yongin 17074, Korea; 5 Department of Chemical Engineering, Hankyung National University, Anseong 17579, Korea; dutuya@hknu.ac.kr

**Keywords:** BRICHOS domain, thermotolerance, chaperone, beta-amyloid (1-42), unfolded protein response

## Abstract

*Caenorhabditis elegans C09F5.1* is a nematode-specific gene that encodes a type II transmembrane protein containing the BRICHOS domain. The gene was isolated as a heat-sensitive mutant, but the function of the protein remained unclear. We examined the expression pattern and subcellular localization of C09F5.1 as well as its roles in thermotolerance and chaperone function. Expression of *C09F5.1* under heat shock conditions was induced in a heat shock factor 1 (HSF-1)–dependent manner. However, under normal growth conditions, most cells types exposed to mechanical stimuli expressed *C09F5.1*. Knockdown of *C09F5.1* expression or deletion of the N-terminal domain decreased thermotolerance. The BRICHOS domain of C09F5.1 did not exhibit chaperone function unlike those of other proteins containing this domain, but the domain was essential for the proper subcellular localization of the protein. Intact C09F5.1 was localized to the Golgi body, but the N-terminal domain of C09F5.1 (C09F5.1-NTD) was retained in the ER. C09F5.1-NTD delayed paralysis by beta-amyloid (1-42) protein (Aβ_42_) in Alzheimer’s disease model worms (CL4176) and activated the unfolded protein response (UPR) by interacting with Aβ_42_. An intrinsically disordered region (IDR) located at the N-terminus of C09F5.1 may be responsible for the chaperone function of C09F5.1-NTD. Taken together, the data suggest that C09F5.1 triggers the UPR by interacting with abnormal proteins.

## 1. Introduction

Organisms recognize acute environmental changes such as high temperature, high salt, oxidizing conditions, or high levels of heavy metals as stress. Some environmental stresses cause denaturation of cellular proteins and accumulation of these denatured proteins is perceived as proteotoxic damage. To minimize this damage, the denatured proteins are degraded or refolded through the unfolded protein response (UPR) or endoplasmic reticulum–associated degradation (ERAD) pathway. Various chaperone proteins are upregulated to alleviate perturbations of protein homeostasis [1]. Heat shock proteins (HSPs) are representative molecular chaperones induced by trimerization of heat shock transcription factor (HSF) following heat shock and other stresses [2,3,4]. Stress proteins can also function as chaperones under normal conditions [5,6]. In general, chaperone proteins assist in proper folding of newly synthesized polypeptides into normal proteins or facilitate the transport of precursor polypeptide chains into appropriate subcellular organelles [7].

The BRICHOS domain, which was initially described in 2002 [8], consists of about 100 amino acids (aa) and is present in more than 300 proteins of 12 distantly related families [9,10]. The designation ‘BRICHOS’ originates from the names of three proteins, which are called BRI2 (Integral transmembrane protein 2B, ITM2B), chondromodulin-I (ChM-I), and surfactant protein-C (SP-C) [8]. All three proteins are linked to major diseases. BRI2 is linked to familial British and Danish dementia (FBD and FDD) [11,12], ChM-I is related to chondrosarcoma [13,14], and SP-C is associated with respiratory distress syndrome (RDS) [15,16]. 

Most BRICHOS domain–containing proteins share common features including type II transmembrane topology and similar domain architectures and subcellular localization patterns [9]. In higher organisms, most of these proteins are proteolytically processed to smaller, functionally mature forms. For example, BRI2, which is well-studied and conserved in vertebrates, is secreted into the extracellular space as the BRICHOS-containing carboxyl-terminal domain following proteolytic processing in the endoplasmic reticulum (ER) and Golgi [17]. BRICHOS domains have similar secondary structures characterized by two well-conserved cysteine residues that form disulfide bridges even though they share little sequence identity at the amino acid level [8].

Three functions of BRICHOS domain–containing proteins have been proposed [8] and proven. These include promotion of targeting and secretion [18,19], assistance with specialized intracellular protease activity [20,21,22,23], and an intramolecular chaperone-like function [18,24,25,26]. Chaperone function is achieved by binding the BRICHOS domain to the β-sheet structural proteins, which prevents the formation of amyloid-like fibrils [10,25,26,27,28,29]. However, there is no known BRICHOS domain-containing protein that is induced by heat shock.

The extracellular region of BRI2, which contains the BRICHOS domain, directly interacts with the amyloidogenic ABri peptide (34 aa), which is generated by a mutation that changes the stop codon to AGA [27]. The BRICHOS domain of BRI2 also efficiently prevents fibrillation of amyloid β-peptide in vivo [30]. Prosurfactant protein-C (proSP-C) expressed in human alveolar type II cells is released to the outside of the cell as a small peptide. Along with phospholipids and various surfactant proteins (SP-A, -B, -D), proSP-C is a component of the pulmonary surfactant that abates surface tension at the interface of air and liquid [31]. Mutations in the BRICHOS domain of proSP-C cause formation of cytosolic aggregates that elicit an ER stress response [32]. The BRICHOS domain of proSP-C also serves as a molecular chaperone to prevent aggregation of amyloid-β peptide [33].

To date, two BRICHOS domain–containing proteins have been identified in *C. elegans*. C09F5.1 is a nematode-specific gene without a human homologue. The other is C25F6.7, which is a homologue of human ITM2B. C09F5.1 is a new type of BRICHOS domain–containing protein whose expression is induced by heat shock. In this study, we characterized the general features of this novel gene and investigate its functions in detail.

## 2. Materials and Methods 

### 2.1. Caenorhabditis elegans and Mammalian Cell Cultures

All *C. elegans* strains were obtained from the *Caenorhabditis elegans* Genetic Center (University of Minnesota, St. Paul, MN, USA). Worms were cultured in nematode growth medium (NGM) at 16 °C or 20 °C as described previously [34]. *N2* Bristol was used as the wild type and transgenic worms were generated by microinjection [35]. The strains used in this study are listed in Table 1.

All mammalian cells used in this study were cultured in Dulbecco’s modified Eagle’s medium (DMEM, D6429) supplemented with 10% fetal bovine serum (FBS, F2442) (SIGMA, St. Louis, MO, USA). For transient expression, cells at 50% confluence were treated with 3 µg of plasmid DNA and 15 µg of 25 kDa linear polyethylenimine (PEI; Polysciences, Warrington, PA, USA) in 150 mM CaCl_2_. One day after transfection, complete medium was added without replacing the medium and the cells were harvested for further analysis after an additional day.

### 2.2. Protein Domain Analysis

The peptide sequence of the transmembrane domain of C09F5.1 was predicted using TMHMM server v. 2.0 [36]. The BRICHOS domain was detected using Motif Scan [37].

### 2.3. RNA Interference

RNA interference by bacterial feeding was performed as described previously [38,39]. Briefly, *HT115(DE3)* was transformed with the RNA interference (RNAi) vector (L4440), which contained part of the *C09F5.1* gene (chrIII:660661 + 661376). Then it was incubated for 4 h at 37 °C in LB with 100 µg/mL ampicillin and 0.4 mM isopropyl β-D-1-thiogalactopyranoside (IPTG) to induce expression of dsRNA. Induced *HT115(DE3)* were seeded onto an RNAi-NGM agar plate with 0.4 mM IPTG and 100 µg/mL ampicillin. Synchronized *pk1426*(*rrf-3*) L1 worms were transferred to each RNAi-NGM plate.

### 2.4. Thermotolerance Assay

Thermotolerance assays were performed as previously described [40] with a slight modification. Synchronized embryos were prepared from eggs collected through treatment of adult worms with alkaline hypochlorite and sodium hydroxide. L2/L3 worms (*n* = 30) were cultured under continuous heat shock (33 °C). Dead worms were counted every 3 h until all the worms were dead. Worms that did not respond to touch with a platinum wire were counted as dead.

### 2.5. Immunoblot Analysis

The C09F5.1 protein was detected by immunoblotting using a standard protocol. Worms were sonicated in lysis buffer (50 mM HEPES pH 7.5, 75 mM sucrose, 6 mM MgCl_2_, 1% NP-40, complete mini protease inhibitor cocktail (Roche, Indianapolis, IN, USA)) and centrifuged at 4 °C at 17,300 *g* for 15 min to remove insoluble materials. Cleared total lysate was quantified by Bradford protein assay (B6916; SIGMA, St. Louis, MO, USA). Twenty micrograms of protein were resolved on 12% sodium dodecyl sulfate polyacrylamide gels and then transferred onto polyvinylidene difluoride (PVDF) membrane using a Mini Trans-Blot Electrophoretic Transfer Cell (Bio-Rad, Philadelphia, PA, USA). Immunoblotting of C09F5.1 was performed with polyclonal rabbit anti-C09F5.1 antibody (GenScript, Piscataway, NJ, USA) diluted 1:5000. Actin (used as a loading control) was detected with anti-β-actin antibody (C4, Santa Cruz Biotechnology, Dallas, TX, USA). Signals were developed using Luminata Forte Western HRP Substrate (Millipore, Billerica, MA, USA). To identify beta-amyloid (1-42) (Aβ_42_) oligomer species in transgenic worms, immunoblotting analysis was performed as described in reference [41]. Briefly, quantified lysates were separated by Tris-tricine SDS-PAGE (13% polyacrylamide) and the PVDF membranes were boiled in PBS for 3 min after the transfer. The blotted membranes were probed with monoclonal mouse anti-Aβ (1-16) antibody (6E10, Covance, Dedham, MA, USA) in 5% nonfat milk. 

### 2.6. Immunoprecipitation of C09F5.1 and Aβ_42_ in 293T Cells

HEK-293T cells transfected with human influenza hemagglutinin (HA)-tagged C09F5.1 variant and Aβ_42_-GFP were sonicated in immunoprecipitation buffer (50 mM Tris-HCl pH 7.5, 150 mM NaCl, 10% glycerol, 1% NP-40, 1 mM EDTA, complete protease inhibitor cocktail (Roche)), and then centrifuged at 17,300 *g* for 10 min at 4 °C to remove insoluble materials. Protein concentrations of cleared lysates were determined by using the Bradford assay. Each lysate (500 µg of total protein) was pre-cleared at 4 °C for 1 h with normal rabbit IgG and protein G resin (P3296; SIGMA). Cleared lysates were incubated with mouse monoclonal anti-HA antibody (F-7, Santa Cruz Biotechnology) at 4 °C for 12 h followed by incubation with protein G resin at 4 °C for 4 h. Immunoprecipitated samples were washed four times with wash buffer (50 mM Tris-HCl pH 7.5, 150 mM NaCl, 1% NP-40, 1 mM EDTA) and mixed with 2× SDS sample buffer. Samples were boiled and analyzed by immunoblotting analysis.

### 2.7. Aβ_42_ Paralysis Assay and Thioflavin T Staining

Synchronized transgenic worms were maintained at 16 °C until the L3 stage and then transferred to 25 °C to induce the expression of Aβ_42_ peptide and C09F5.1 variants in body wall muscle cells. Paralyzed worms, which are defined as those that did not roll over when touched with a platinum wire pick, were scored every day until all the worms were paralyzed. Thioflavin T staining was performed to detect Aβ_42_ aggregates in the body wall muscle. Samples prepared for the Aβ_42_ paralysis assay were washed with M9 and fixed with 4% formaldehyde (in PBS) at 4 °C for 24 h. The fixed samples were permeabilized overnight at 37 °C in 125 mM Tris-HCl (pH 7.5), 1% Triton X-100, and 5% β-mercaptoethanol. Then the samples were stained with 0.125% thioflavin T (SIGMA) in 50% ethanol for 2 min. The worms were de-stained with sequential ethanol washes (50%, 75%, 90%, 75%, and 50% *v*/*v*), washed with M9 buffer containing 1% Triton X-100, and then mounted on slides for microscopy. Fluorescence images of amyloid aggregates in the body wall muscles were obtained by confocal microscopy (DM4000; Leica, Wetzlar, Germany).

### 2.8. Detection of UPR Activity Using the ERAI System

UPR activity was detected in HEK-293T transfected with the ERAI system. pERAI plasmid DNA was constructed by using molecular cloning techniques as previously described [42] with slight modifications. The FLAG-tagged xbp1ΔDBD fragment was fused with the mVenus coding sequence in the pCAX backbone plasmid to yield pCAX-ERAI. HEK-293T cells were transfected with pCAX-ERAI and selected with G418. To confirm that the ERAI system functioned properly, tunicamycin was used as a positive control for inducing ER stress. The degree of UPR activation was determined by measuring the amount of XBP1ΔDBD-mVenus in immunoblots with the anti-GFP antibody.

### 2.9. Subcellular Localization of C09F5.1 Variants in COS7

All plasmid DNA used in this experiment was prepared by molecular cloning. mCherry fused with C-terminal KDEL was used as an ER marker [43,44] and mCherry with the Golgi targeting sequence of B4GALT1 was used as a Golgi marker [45]. Plasmids containing C-terminal GFP–fused C09F5.1-FL, N-terminal domain (-NTD), C-terminal domain (-CTD), or -C455S and the subcellular localization marker were co-transfected into COS7 cells using PEI. Two days after transfection, fluorescence images were obtained on a confocal microscope equipped with a CCD camera.

## 3. Results

### 3.1. Characteristics of C09F5.1

**Figure 1:**
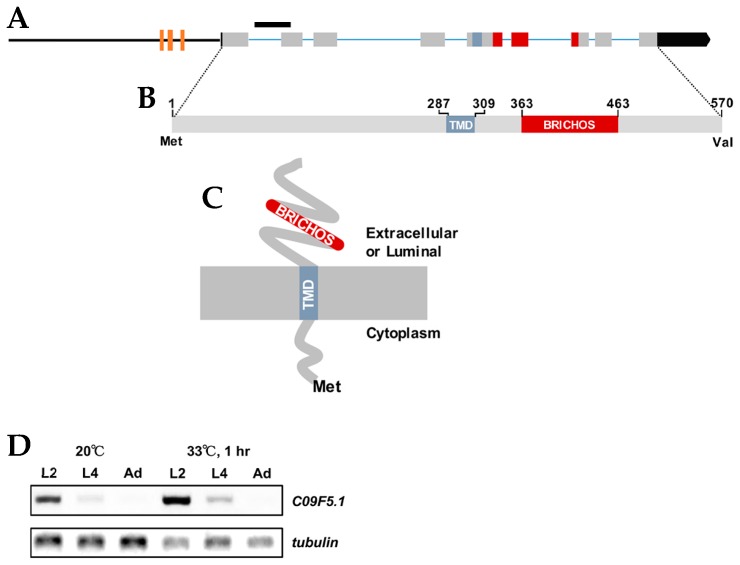
C09F5.1 gene and its encoded protein. (**A**) *C09F5.1* gene structure. The black bar indicates the deletion site in the *C09F5.1(ok2863)* mutant VC2139 strain. Brown boxes indicate heat shock elements (HSEs) (nGAAnnTTCn) at nucleotides −964 to −950 (cGAAaaTTCcaaac), −950 to −925 (tGAAttTTCtccaaa), and −730 to −721 (cGAActTTCg). (**B**) C09F5.1 protein. TMD: transmembrane domain. (**C**) Predicted membrane topology of C09F5.1 protein. (**D**) Transcript levels of *C09F5.1*. Total RNA was isolated from synchronized *N2* at each growth stage under normal growth temperature or heat stress. The reverse transcription (RT)-PCR product of total RNA was amplified with C09F5.1-specific primers. β-tubulin was used as an internal loading control. TMD: Transmembrane domain.

The *C09F5.1* gene was isolated by differential-display reverse transcription–PCR (ddRT-PCR) between wild-type N2 and a heat-sensitive mutant. The gene contains nine exons and encodes a predicted type II membrane protein of 570 aa including a 286 aa N-terminal cytosolic region, a 23 aa transmembrane domain, a 101 aa BRICHOS domain, and a 107 aa C-terminal region (Figure 1A–C). The *C09F5.1* promoter contains three heat shock elements (HSEs; nGAAnnTTCn) at nucleotides −964 to −950 (cGAAaaTTCcaaac), −950 to −925 (tGAAttTTCtccaaa), and −730 to −721 (cGAActTTCg), which implies that it is induced by the heat shock response. As expected, the transcript level of *C09F5.1* was stimulated by 33 °C heat shock treatment with the highest expression at stages L2 and L4 (Figure 1D). Proteins with high identity (78–86%) to C09F5.1 were found in six *Caenorhabditis* species, but no protein orthologue was found in non-nematode phyla. Multiple sequence alignment analysis between C09F5.1 and other BRICHOS domain-containing protein family members revealed that the C09F5.1 family constitutes a separate group and therefore represents a distinct subfamily (Supplementary Figure S1). In addition, when the amino acid sequences of C09F5.1 homologues are divided into the N- and C-termini, the amino acid identity was found to be higher in the C-terminal than in the N-terminal region, which was determined by ClustalW (Supplementary Table S1). The two cysteine residues conserved in BRICHOS domains are present (Supplementary Figure S2A) and two of the three exons encoding the BRICHOS domain were almost identical in Nematoda (Supplementary Figure S2B), which implies that the BRICHOS domain of C09F5.1 is evolutionarily well-conserved.

### 3.2. Expression of C09F5.1 and Localization of Its Product In Vivo

**Figure 2:**
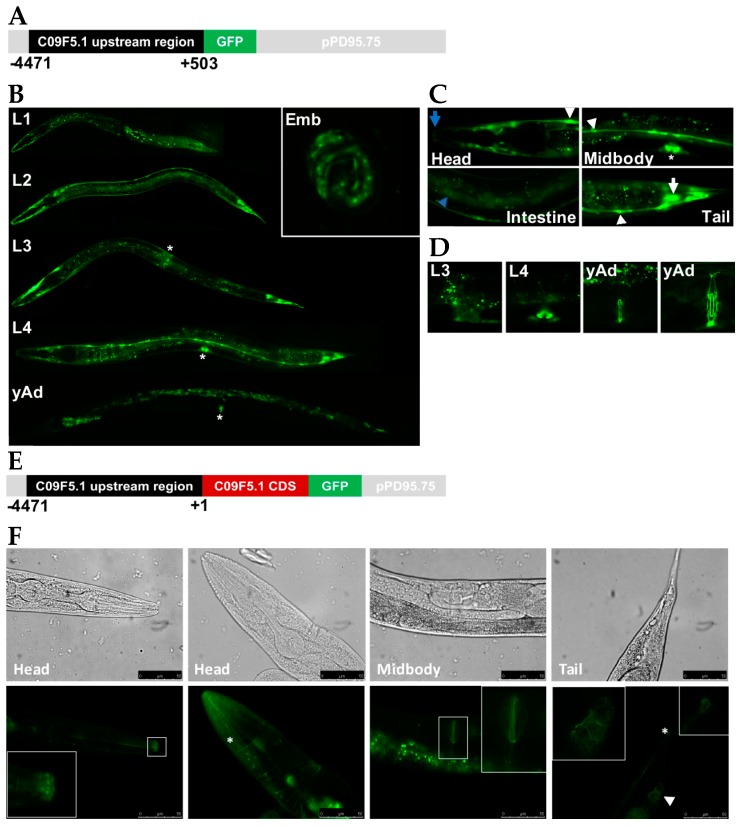
*C09F5.1* expression and localization during *C. elegans* development. (**A**) *C09F5.1 promoter::GFP* reporter construct. The upstream region of *C09F5.1* (−4471–+503) was cloned from N2 genomic DNA and fused with GFP. (**B**) Temporal expression pattern of *C09F5.1 promoter::GFP*. GFP was detected by fluorescence microscopy throughout development from embryo to adult worm. Developmental stages are displayed on the upper left. Asterisk indicates the vulva. Emb: embryo. (**C**) Spatial expression patterns of *C09F5.1 promoter::GFP*. GFP was expressed in the sensillar region (Head, blue arrow), seam cells (Head, Midbody and Tail, arrowhead), vulval cells (Midbody, asterisk), intestinal cells (Intestine, blue arrow head), and a neuron-like cell (Tail, white arrow) of a L4-stage worm. (**D**) Temporal expression pattern of *GFP* in the vulval region. Developmental stages are indicated on the upper left. yAd: young adult. (**E**) C09F5.1-GFP fusion construct. The putative promoter region of *C09F5.1* (−4471–+1) was ligated to the *C09F5.1* cDNA sequence and subcloned into pPD95.75 (promoter-less GFP reporter vector) to yield a GFP fusion protein. (**F**) Spatial expression pattern of C09F5.1-GFP fusion protein. Wild-type N2 was transformed with *C09F5.1p::C09F5.1::GFP* and the *rol-6* (su1006) selection marker by microinjection. GFP was detected in young adult worms by fluorescence microscopy. C09F5.1-GFP was localized at the sensillar region (Head, white square box), head neurons (Head, asterisk), the vulval junction (Midbody, white square box), the tail neuron (Tail, asterisk), and the junction of cells around the sphincter muscle (Tail, arrowhead). Inserts show enlargements of boxed areas. CDS: Coding sequence.

To elucidate the physiological function of C09F5.1, we studied the spatiotemporal expression pattern of *C09F5.1* in *C. elegans* using a *GFP* reporter. About 5 kb of the upstream region (from -4471 bp upstream to +503 bp downstream of the transcription initiation site) is expected to contain the *C09F5.1* promoter, which was cloned from genomic DNA and subcloned into pPD95.75 known as a promoter-less GFP expression vector (see Figure 2A). Expression of *GFP* under the control of the *C09F5.1* promoter was observed through all developmental stages (see Figure 2B). In 3-fold embryos, GFP was localized in seam cells and hypodermal cells (see Figure 2B, Emb). 

In the larval stage, strong expression was detected in the sensillar region (lip) (Figure 2C, Head), seam cells, vulval cells (Figure 2C, Midbody), intestinal cells (Figure 2C, Intestine), and tail neurons (Figure 2C, Tail). Expression in the hypodermal cells gradually decreased as the worms progressed to L3 and L4 and almost no expression was detected in the hypodermal cells of young adults (Figure 2B, L3, L4, and yAd). During vulva formation, GFP was detected in a subset of vulval cells (mainly vulA) (Figure 2D, L4) in L4. After completion of vulval development, overall GFP signal in the vulval region decreased and only persisted at the vulval junction (Figure 2D). These data suggest that C09F5.1 may be involved in developing and forming the vulva. This hypothesis is supported by a previous observation that RNAi knockdown of *C09F5.1* in *pk1426(rrf-3)* results in a protruding vulva phenotype [46].

Because transcriptional fusion may not reflect the true expression pattern of the gene, we also generated a transgenic worm expressing *C09F5.1-GFP* under the control of the *C09F5.1* promoter. For this purpose, *C09F5.1* cDNA was subcloned into pPD95.75 containing the upstream region of *C09F5.1* (−4471–+1) (Figure 2E), and then injected into gonad of wild-type N2. Even though *GFP* expression in the early larva stage (L1–L3) was lower for the fusion construct than the promoter-GFP construct described above, GFP expression in this transgenic worm exhibited patterns similar to those observed in *P_C09F5.1_::GFP*. *C. elegans* has six symmetrical ‘lips’ surrounding the mouth [47] and the sensillar region contains various neurons (six IL1s, six IL2s, two OLLs, four OLQs, and cephalic neurons) that play general roles in mechanosensation or chemosensation [48]. C09F5.1-GFP was localized in the six sensillar lips and in the head region in a neuron-like pattern (Figure 2F, Head). These results suggested that C09F5.1 might function in the sensory organs. In addition, C09F5.1-GFP was detected in the vulval junction (Figure 2F, Midbody) and the tail neuron (Figure 2F, Tail), which was also observed in the *P_C09F5.1_:GFP* transgenic worm.

### 3.3. C09F5.1-Dependent Thermotolerance and HSF-1-Dependent C09F5.1 Expression 

**Figure 3:**
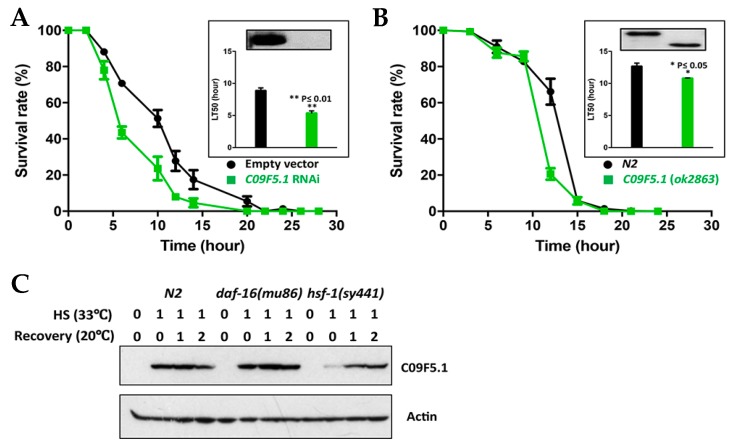
Contribution of C09F5.1 to thermotolerance, and HSF-1–dependent expression of *C09F5.1*. (**A**,**B**) Thermotolerance assay. Synchronized *rrf-3*(*pk1426*) were fed bacteria expressing *C09F5.1* dsRNA. Worms grown to the L3 stage were exposed to 33 °C and observed for movement every 3 hr. The *C09F5.1*(*ok2863*) mutant was also tested in the same manner. The insert in the graph shows an immunoblot for C09F5.1 in each worm along with the median Lethal Time (LT_50_) value. Data represent means ± SEM (20 worms/plate, three plates). Three independent experiments were performed and yielded consistent results. (**C**) C09F5.1 expression in transcription factor mutants and protein levels during the recovery period. Synchronized L3 worms were recovered at 20 °C after exposure to 33 °C for the indicated times. Worm lysates prepared from each strain were quantified by the Bradford assay. Equal amounts of protein were separated by Tris-glycine SDS-PAGE and immunoblotted with anti-C09F5.1 antibody. Anti-β-actin antibody was used to detect actin (loading control). dsRNA: Double-strand RNA; RNAi: RNA Interference; SEM: Standard error of the mean; HSF: Heat shock transcription factor.

Because *C09F5.1* transcription was induced by heat shock and the gene was identified in a heat-sensitive mutant, we performed thermotolerance assays on L2/L3 worms at 33 °C. Worms in which C09F5.1 was knocked down by bacterial feeding of RNAi were more sensitive to heat shock (33 °C) than control worms harboring the empty vector (LT_50_(Con/Exp) = 1.66) (Figure 3A). In addition, worms with a deletion mutation in *C09F5.1*(*ok2863*) also died sooner than wild-type N2 under heat shock conditions (33 °C) (LT_50_(wt/mt) = 1.17) (Figure 3B). This shows that functional inactivation of *C09F5.1* increases sensitivity to heat shock. However, the sensitivity was slightly lower in the deletion mutant, which suggests that other regions of the C09F5.1 protein were not affected by the deletion contribution of thermotolerance. 

In *C. elegans*, induction of molecular chaperone proteins under various types of cellular stress is primarily regulated by HSF-1, a FOXO-family transcription factor (TF; DAF-16), or a Nrf family TF (SKN-1) [49,50]. Because SKN-1 regulates the oxidative stress response [51,52] and does not respond to heat shock treatment [53], we did not study it in the following experiment. To determine which TF induces the expression of *C09F5.1* upon heat shock, we analyzed the expression pattern of *C09F5.1* in worms harboring mutations in HSF-1 or DAF-16 (see Figure 3C). In both wild-type N2 and the *daf-16*(*mu86*) mutant, expression of *C09F5.1* was immediately induced after heat shock at 33 °C for 1 hr and the protein levels gradually decreased as the worms recovered (see Figure 3C). By contrast, in the *hsf-1*(*sy441*) mutant, induction of *C09F5.1* gene expression was delayed, which suggests that *C09F5.1* expression depends on the HSF-1 pathway and that C09F5.1 has a chaperone function related to thermotolerance but not to other stresses.

### 3.4. N-Terminal-Dependent Interaction of C09F5.1 with Aβ_42_

**Figure 4:**
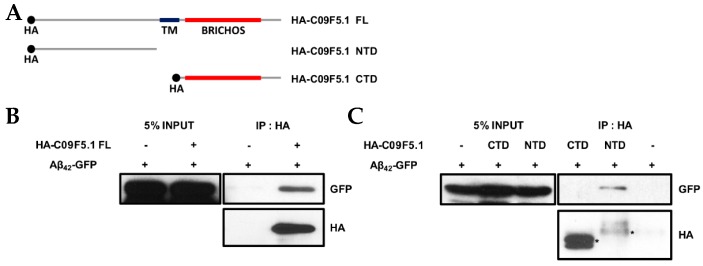
N-terminus–dependent interaction of C09F5.1 with Aβ_42_. (**A**) HA-fusion constructs of C09F5.1 used in the immunoprecipitation assay. (**B**, **C**) Immunoprecipitation assay of 293T cells expressing HA-fused C09F5.1 variants and Aβ_42_-GFP. Cell lysates prepared from transfected 293T cells were quantitated by the Bradford assay. Equal amounts of proteins were used (shown as 5% input) for immunoprecipitation assays. Asterisks indicate immunoprecipitated proteins. CTD: C-terminal domain; NTD: N-terminal domain; HA: Human influenza hemagglutinin.

Because the chaperone function of BRICHOS domain-containing proteins [27,54,55] reduces amyloid toxicity by inhibiting amyloid fibril formation [33,56], we examined the chaperone function of C09F5.1 in mammalian cells. First, we demonstrated that C09F5.1 interacts with amyloid-β peptide in co-transfected HEK-293T cells (Figure 4B). To assess the role of the BRICHOS domain in chaperonin function, we dissected C09F5.1 into two parts: The two parts are the cytosolic NTD and the CTD, which contains the BRICHOS domain. Unexpectedly, the C-terminal domain did not interact with beta-amyloid (1-42) protein (Aβ_42_)-GFP whereas the N-terminal domain did (see Figure 4C).

### 3.5. Amyloid Toxicity Assay in C. elegans Alzheimer’s Disease Model Expressing C09F5.1-NTD

**Figure 5:**
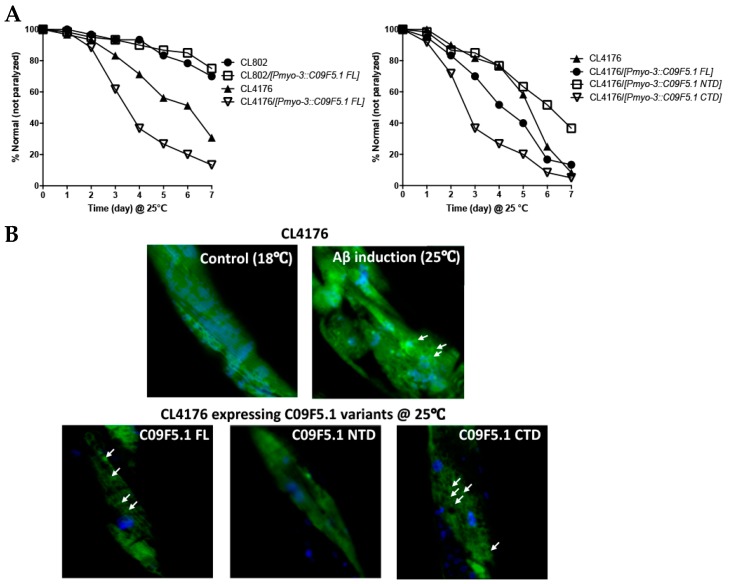

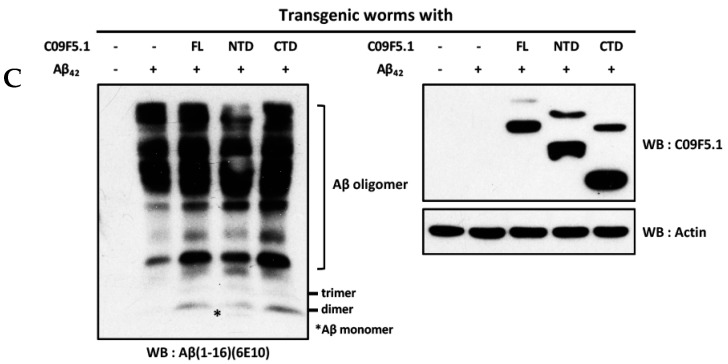
Cytotoxicity test of Aβ_42_ in Alzheimer disease model worms expressing C09F5.1 variants. (**A**) Aβ_42_ paralysis assay. Plasmid DNA encoding *C09F5.1* (-FL, -NTD, or -CTD) driven by the *myo-3* promoter was microinjected into CL4176 with pBCN41 (G418 resistance gene). All transgenic worms were selected by G418. L2/L3 worms synchronized at 16 °C and were temperature-shifted to 25 °C to allow accumulation of Aβ_42_ peptide. The number of paralyzed worms was counted daily. Twenty worms per plate and three plates per experiment were used for each transgenic line. Three independent experiments were performed and yielded consistent results. CL802 was used as a negative control for CL4176. (**B**) Thioflavin T staining assay. Worms prepared in the same manner as for the Aβ_42_ paralysis assay were harvested three days after the growth temperature was increased to 25 °C. Worms stained with thioflavin T (green) and DAPI (nuclei; blue) were analyzed by confocal microscopy. CL4176 grown at a permissive temperature (18 °C) was used as a negative control. All images were obtained by focusing on the body wall muscle in the middle of the body. White arrows indicate Aβ_42_ deposits. (**C**) Detection of Aβ_42_ oligomer species. Lysates were obtained from transgenic worms that had accumulated Aβ_42_ for 3 days and proteins were quantified by Bradford assay. Equal amounts of protein were separated by Tris-tricine SDS-PAGE (13%) with MES running buffer and immunoblot assays were performed. Aβ_42_ oligomers were detected with anti-Aβ antibody (6E10) and C09F5.1 variants were detected with anti-HA antibody. Anti-β-actin antibody was used to detect actin (loading control). WB: Western blot.

In light of the interaction between the N-terminal domain of C09F5.1 (C09F5.1-NTD) and Aβ_42_ peptide in 293T, we further examined the relationship between these proteins in a *C. elegans* Alzheimer’s disease model [57] by performing amyloid toxicity tests. For this purpose, we generated transgenic worms derived from CL4176 (*smg-1(cc546 ts) I; dvIs27 [myo-3/Aβ_42_ minigene + rol-6(su1006)]*) expressing HA-tagged C09F5.1s (-FL, -NTD, or -CTD) in the body wall muscles. Synchronized transgenic worms were maintained at 16 °C until stage L2/L3 and then the temperature was increased to 25 °C (non-permissive temperature) for paralysis assays. Transgenic worms co-expressing Aβ_42_ and C09F5.1-NTD were more resistant to Aβ_42_ toxicity than CL4176 (parental strain) or worms co-expressing C09F5.1-CTD and Aβ_42_. Transgenic worms co-expressing Aβ_42_ and C09F5.1-FL were more severely paralyzed than CL4176. Paralysis of transgenic worms was not related to the accumulation of C09F5.1 itself (see Figure 5A). We then performed the thioflavin T staining assay to determine whether the delayed paralysis in transgenic worms co-expressing Aβ_42_ and C09F5.1-NTD was due to a reduction in the amount of Aβ_42_ oligomer. We observed lower levels of thioflavin T–positive aggregates in worms co-expressing Aβ_42_ and C09F5.1-NTD than in those expressing C09F5.1-FL or C09F5.1-CTD, which exhibited disorganization of actin filaments in the body wall muscle (see Figure 5B). Immunoblot analysis of worm lysates with the anti-Aβ antibody also revealed relatively low levels of Aβ_42_ oligomers in worms expressing C09F5.1-NTD (Figure 5C). This was similar to the results of the thioflavin T assay. These observations indicated that C09F5.1-NTD might be involved in decreasing toxicity of Aβ_42_ oligomers by binding to Aβ_42_. However, in contrast to previously reported BRICHOS domain-containing proteins in other species, the BRICHOS domain of C09F5.1 did not have chaperone activity.

### 3.6. Subcellular Localization and Unfolded Protein Response

**Figure 6:**
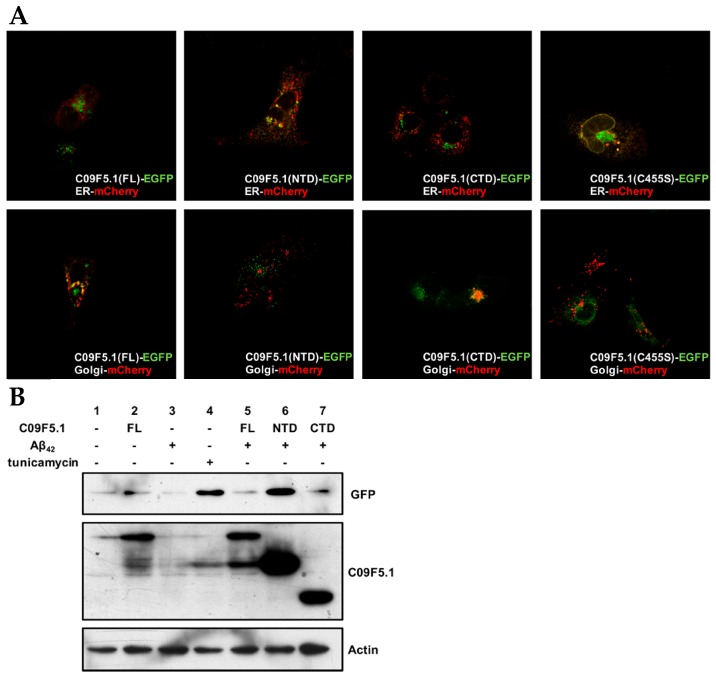
Subcellular localization of C09F5.1 and UPR activation by Aβ_42_ in the ER. (**A**) Subcellular localization of truncated C09F5.1 variants transiently expressed in COS7 cells. COS7 cells were co-transfected with GFP-fused C09F5.1 and ER or Golgi-mCherry marker. All images were acquired using a confocal microscope equipped with a coupled-charge device (CCD) camera. (**B**) Activation of the unfolded protein response (UPR). The ERAI stable cell line was constructed by transfecting 293T cells with pERAI plasmid containing xbp1ΔDBD-mVenus. The cells were treated for 5 h with 5 µg/mL tunicamycin or DMSO (vehicle control) for 36 h after transfection. All cells were harvested at the same time and cell lysates were quantified by the Bradford assay. Equal amounts of protein were separated by the Tris-glycine SDS-PAGE system and immunoblotted with anti-GFP and anti-HA antibodies. Anti-β-actin antibody was used as a loading control. ER: Endoplasmic Reticulum

Because one of the putative functions of the BRICHOS domain is to assist in targeting and secreting proteins [8], we examined the subcellular localization of GFP fusion proteins in COS7 cells (see Figure 6A). C09F5.1-NTD without the BRICHOS domain was mainly detected in the ER whereas both C09F5.1-FL and C09F5.1-CTD (which possess the BRICHOS domain) were observed largely in the Golgi. Mutation of one of the two conserved cysteine residues in the BRICHOS domain to serine (C455S) caused the GFP signal to accumulate in the ER. These results suggested that the intact BRICHOS domain including the two conserved cysteine residues are essential for normal trafficking of C09F5.1. 

In CL4176, an Alzheimer’s disease model of *C. elegans*, abnormal human Aβ_42_ proteins are retained in the ER and retrotranslocated into cytoplasm via an interaction between Aβ_42_ and ER chaperones [58]. The reduction of Aβ_42_ toxicity (see Figure 4C) and ER localization of C09F5.1-NTD (see Figure 6A) suggest that C09F5.1-NTD might be related to the ER stress response. To investigate the relationship between C09F5.1 and the UPR, we used the ER stress–activated indicator (ERAI) system in mammalian cells [42]. First, we transfected ERAI stable cells with the indicated constructs and then measured the level of UPR activity in each sample by monitoring the level of *XBP1*ΔDBD-mVenus protein. Treatment with an ER stress inducer, tunicamycin, was used as a positive control. When C09F5.1-FL, -NTD or Aβ_42_ was expressed alone, the ER stress level was similar to that in the negative control (Figure 6B, lanes 2, 3; Supplementary Figure S3). On the other hand, when C09F5.1-NTD was co-expressed with Aβ_42_, UPR activity was similar to that in the positive control group (Figure 6B, lanes 4, 6). Interestingly, the morphology of cells treated with tunicamycin changed drastically whereas the morphology and viability of cells co-expressing C09F5.1-NTD and Aβ_42_ did not (data not shown). When C09F5.1-FL or the C-terminal region containing the BRICHOS domain (C09F5.1-CTD) was co-expressed with Aβ_42_, the UPR was activated very little or not at all (Figure 6B, lanes 5, 7). Because the C09F5.1-NTD interacts directly with Aβ_42_ and is localized in the ER and the expression of C09F5.1-NTD reduced the rates of paralysis in these worms (Figure 5A), these findings indicate that the complex of C09F5.1-NTD and Aβ_42_ activated the UPR, which decreased the cytotoxicity of the Aβ_42_ oligomer.

## 4. Discussion

The BRICHOS domain is present in many proteins from a wide range of species [8,9,10]. Most BRICHOS domain–containing proteins consist of a cytosolic N-terminal domain, a hydrophobic TM region, a linker region followed by the BRICHOS domain, and a C-terminal region. All of these entities are considered to be type II membrane proteins. The *C09F5.1* gene identified by ddRT-PCR in a heat-sensitive mutant encodes a novel protein belonging to the BRICHOS family (Figure 1; Supplementary Figures S1 and S2). The similarity between this protein and other homologues at both the genomic DNA and protein structure levels suggested that C09F5.1 plays roles similar to those previously proposed for this protein family such as assistance in the secretion pathway, cellular protease activity, and chaperone-like functions.

The expression pattern and subcellular localization of a gene product give indirect information about the function of a gene. We found that the C09F5.1 protein was expressed in junctions between vulval toroids, junctions of cells surrounding sphincter muscle, and the six symmetrical lips of the sensillar region (see Figure 2). Because these are all sites where cells are constantly exposed to external environmental signals and stresses, the intercellular interactions in these regions must be tightly regulated. In particular, the spatial expression pattern of *C09F5.1* was supported by a previous report that *C09F5.1* knockdown causes a protruding vulva phenotype [46]. Gastrokine-1, a BRICHOS domain-containing protein, increases the tight junction proteins to maintain the integrity of intercellular junctions [59]. In addition, c-Jun N-terminal Kinase (JNK), a stress-activated protein kinase that is expected to phosphorylate Thr-280 in C09F5.1 (Supplementary Table S2) is also involved in regulating intercellular adhesion [60]. Therefore, C09F5.1 might play a role in regulating the integrity of cells under stress or changing environmental conditions. Based on the results shown in Figure 3, it is likely that C09F5.1 is involved in temperature stress responses.

Because one of the functions of BRICHOS domain–containing protein is chaperone-like activity, we examined the effects of C09F5.1 on cytotoxicity in mammalian cells (see Figure 4) and a *C. elegans* Alzheimer’s disease model (see Figure 5). However, we found that the BRICHOS domain of C09F5.1 (represented by C09F5.1-FL or C09F5.1-CTD) had no apparent chaperone function. Specifically, we observed no decrease in Aβ_42_-induced paralysis in CL4176 worms expressing the BRICHOS domain of C09F5.1. On the other hand, Aβ_42_-induced paralysis was delayed by co-expression of C09F5.1-NTD in CL4176 worms. In the presence of C09F5.1-NTD, Aβ_42_ induced ER stress, which activates the UPR, we detected an increase in alternative splicing of *xbp-1* (see Figure 6B). However, this stress did not result in cell death (data not shown). Thus, C09F5.1-NTD might possess a chaperone-like function that decreases amyloid cytotoxicity by inhibiting fibril formation. The chaperone proteins accumulated following UPR activation might prevent Aβ_42_ from being turned to toxic aggregates or alternatively decrease Aβ_42_ synthesis by downregulating overall translation.

BRICHOS domain-containing proteins act as chaperones by binding to proteins with extensive β-sheet structure and preventing formation of amyloid-like fibrils [10,25,26,27,28,29]. The chaperone functions of BRICHOS domain-containing proteins such as ChM-I, BRI2, and CA11 depend on the cysteine-rich C-terminus [24,28] whereas that of lung SP-C resides in its 35 aa transmembrane domain [24,26]. However, the chaperone function of C09F5.1 resided in its N-terminal domain. In the *C09F5.1(ok2863)* mutant, which was more sensitive to heat shock than the wild-type N2, the C09F5.1 protein contains a deletion in the N-terminal region and lacks aa 76–137. Interestingly, this region overlaps almost completely with the intrinsically disordered region (IDR) in the N-terminus of C09F5.1. More than half of eukaryotic proteins have IDRs that do not form well-structured domains due to the lack of large hydrophobic amino acids (I, L, and V) [60,61,62,63]. The biological functions of IDRs have been classified into 28 distinct categories by Dunker and colleagues based on previous studies [64]. Broadly, these functional categories encompass molecular recognition, molecular assembly, protein modification, and entropic chain activities [63]. IDRs interact with macromolecules weakly and transiently, but more rapidly in comparison with well-organized domains [65,66,67]. This dynamic characteristic is effective for inhibiting aggregation or solubilization of protein aggregates [63]. These facts suggest that the C09F5.1 NTD slows down oligomerization by interacting with the Aβ_42_ monomer through its disordered region. Consistent with this idea, Aβ_42_ monomers were only observed in transgenic worms expressing C09F5.1-NTD (Figure 5C). Also, IDRs are easily accessible to small molecules or proteins and therefore help to ensure specific post-translational modifications (PTMs) associated with protein stability and subcellular localization [68,69]. Indeed, the IDR deleted in *C09F5.1(ok2863)* covers the N-glycosylation site and GSK3 phosphorylation recognition sites as well as Ser-83 targeted by c-JNK, which is likely to play an important role in thermotolerance. However, further studies are needed to determine how these PTMs influence thermotolerance.

Another proposed function of the BRICHOS domain-containing proteins is assistance in secretory transport [8]. Two major BRICHOS domain–containing proteins, BRI2 and proSP-C, are transported through the ER and Golgi to the plasma membrane and multi-vesicular bodies [12,70]. Deletion of a part of the BRICHOS domain of SP-C prevents transport to the Golgi [32] and altering certain amino acids in the BRICHOS domain of proSP-C guides the mutant protein to various intracellular locations [71]. Likewise, when one of the two cysteines in the BRICHOS domain of C09F5.1 was replaced with a serine, the mutant protein was retained in the ER whereas intact the C09F5.1-CTD–GFP fusion protein was localized in the Golgi (see Figure 6A). Therefore, although the BRICHOS domain of C09F5.1 does not interact with Aβ_42_ or function as a chaperone, it is essential for the proper subcellular localization of the protein.

## Figures and Tables

**Table 1:** List of *Caenorhabditis elegans* strains used in this study.

Strain ^a^	Background Genotype	Transgene
N2	*C. elegans* wild type	
VC2139	*C09F5.1(ok2863) III*	
PS3551	*hsf-1(sy441) I*	
CF1038	*daf-16(mu86) I*	
NL2099	*rrf-3(pk1426) II*	
CL802	*smg-1(cc546) I; rol-6(su1006) II*	
CL4176	*smg-1(cc546) I; dvIs27 X* ^b^	
MC9220	*smg-1(cc546) I; rol-6(su1006) II*	*[myo-3p::HA-C09F5.1 FL::let-858 3′UTR, myo-2p::gfp(S65C)*, *rpl-28p::neoR]*
MC9221	*smg-1(cc546) I; dvIs27 X*	*[myo-3p::HA-C09F5.1 FL::let-858 3′UTR, myo-2p::gfp(S65C)*, *rpl-28p::neoR]*
MC9222	*smg-1(cc546) I; dvIs27 X*	*[myo-3p::HA-C09F5.1 NTD::let-858 3′UTR, myo-2p::gfp(S65C)*, *rpl-28p::neoR]*
MC9224	*smg-1(cc546) I; dvIs27 X*	*[myo-3p::HA-C09F5.1 CTD::let-858 3′UTR, myo-2p::gfp(S65C)*, *rpl-28p::neoR]*
MC9226	*smg-1(cc546) I; dvIs27 X*	*[myo-3p::gfp(S65C)::let-858 3′UTR, myo-2p::gfp(S65C)*, *rpl-28p::neoR]*
KH0001	*N2*	*[C09F5.1p::gfp(S65C)::unc-54 3′UTR, rol-6(su1006)]*
MC0001	*N2*	*[C09F5.1p::C09F5.1::gfp(S65C)::unc-54 3′UTR, rol-6(su1006)]*

^a^ All strains were obtained from *C. elegans* Genetic Center (CGC) except that the transgenic lines named KH- or MC- were produced in our laboratory. ^b^ dvIs27 [myo-3p::Aβ_42_::let-858 3′UTR] + rol-6(su1006)]. UTR: Untranslated region. HA: Human influenza hemagglutinin.

## Supplementary Materials

The following are available online at http://www.mdpi.com/2073-4425/9/3/160/s1, Figure S1. Neighbor-joining unrooted phylogenetic tree of BRICHOS domain-containing proteins, Figure S2. High conservation of BRICHOS domain of C09F5.1 in Nematoda, Figure S3. UPR activation by C09F5.1-NTD in ERAI-293T cells, Table S1. Amino acid identity between C09F5.1 nematode homologues and ceC09F5.1, Table S2. Phosphorylation site prediction of C09F5.1 by c-Jun N-terminal kinases.
