# Supplementary material for: Caenorhabditis elegans BRICHOS Domain–Containing Protein C09F5.1 Maintains Thermotolerance and Decreases Cytotoxicity of Aβ42 by Activating the UPR
Source: Genes (Basel). 2018 Mar 13;9(3):160. doi: 10.3390/genes9030160 (PMC5867881; doi:10.3390/genes9030160)
Supplement: Supplementary file 1 [file genes-09-00160-s001.pdf]

Supplementary Materials

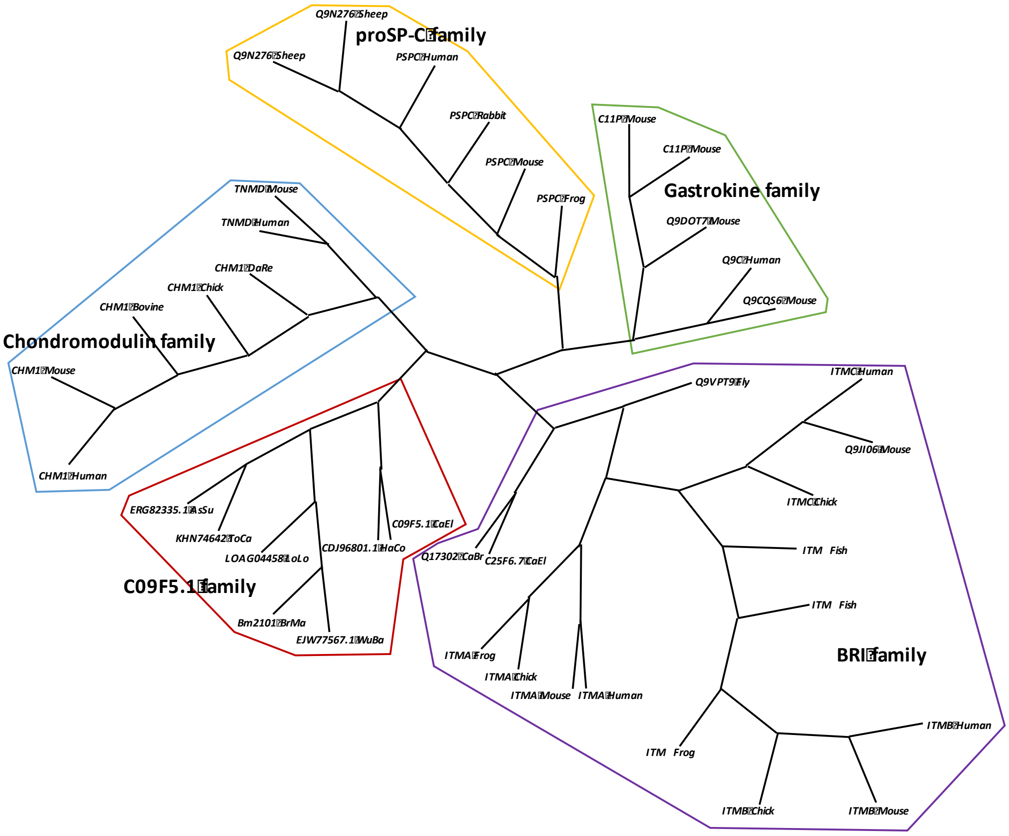

Figure S1. Neighbor-joining unrooted phylogenetic tree of BRICHOS domain-containing proteins. The unrooted phylogenetic tree was constructed by aligning the amino acid sequence of BRICHOS domains using ClustalW (http://www.genome.jp/tools-bin/clustalw). Colors represent each BRICHOS family; the C09F5.1 family is in red. *CaEl*: *C. elegans*, *CaBr*: *C. briggsae*, *HaCo*: *Haemonchus contortus*, *LoLo*: *Loa loa*, *WuBa*: *Wuchereria bancrofti*, *ToCa*: *Toxocara canis*, *BrMa*: *Brugia malayi*, *AsSu*: *Ascaris suum*, *DaRe*: *Danio rerio*.

A

```
LOAG_03358_LoLo      FKSNSSSVIDFETGYIAIADHALIDSSGKHIVCFLLPLDRSAIPSVTALRDALSSVTSEI
EJW77567.1_WuBa      FRSNSFSVIDFETGYVAVADHALIDSSGKQIVCFLIPLDRSAIPNISALRDALSSVTSEI
Bm2101_BrMa          FRSNSLSVIDFETGYVAIADHALIDSSGKQIVCFLIPLDRSAIPNISALRDALSSVTSEI
KHN74642.1_ToCa      FKSNSFTAIDFSTGYIAVADHALTDSQGKHTTCFIMPLDRSAIPSMSALQDALSSSGSEV
ERG82335.1_AsSu      FKSNSFTAIDFNTGYIAIADHALTDSHGKHTTCFVMPLDRTAMPSMSALQDALRSTESEV
C09F5.1_CaEl         FKSNSFTAVDFNTGYVAYADHSLTDANGKHFTCFLMPLDRSAIDSIDQLSEAVSESSYEI
CDJ96801.1_HaCo      FKSNSFTAIDFETGYIAVADHALTDSNGRHFTCFIMPLDRSAIPSMEALREAVSESDYEI
                     *:*** :.:**.***:* ***:* *: *:: .**::****:*: .:  * :*: .   *:

LOAG_03358_LoLo      YSEYGWQEYWQYHAEAIDARSAERKFTNKIDDCRSAKWYL
EJW77567.1_WuBa      YSEYGWQEYWQYHAEAIDAKSAKHKFTNEIDNCQSAKWYL
Bm2101_BrMa          YSEYGWQEYWQYHAEAIDAKSAKRKFTNEIDNCQSAKWYL
KHN74642.1_ToCa      QSEFGWQEYWQYQAEPIDALSAQRKFTDTIADCVNAKWYM
ERG82335.1_AsSu      QSEFGWQEYWQYQAEPIDALSAERKFTERIADCINAKWYL
C09F5.1_CaEl         QSTFGWQEFYQFDPEKIEPMTAKQKFTEEIDDCEGAQWYL
CDJ96801.1_HaCo      QAQFGWQEFWQFDAEPIEPVAANSKFTERISDCVGAKWY-
                      : :****::*:. * *:  :*: ***: * :* .*:**
```

B

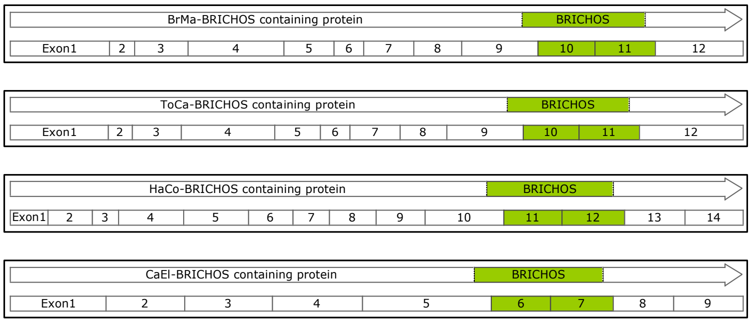

Figure S2. High conservation of BRICHOS domain of C09F5.1 in Nematoda. (A) Multiple sequence alignment of the BRICHOS domain of the C09F5.1 family. The BRICHOS domain of the C09F5.1 family was subjected to multiple sequence alignment using Clustal Omega. An asterisk indicates the same amino acid, and a colon and a period indicate degrees of similarity. The gray highlighting represents two cysteine residues that must be present in the BRICHOS domain. (B) Comparison of exon structure of C09F5.1 homologues. The exon structure and amino acid sequence of four C09F5.1 homologues in the Nematoda phylum were labeled with the BRICHOS domain using SnapGene. In the black box, the upper white arrow represents protein and the lower white block represents coding sequence. The number in the block is the exon number and the green labeling indicates the location of the BRICHOS domain. The black line on the left represents the genetic distance of the BRICHOS domain sequence.

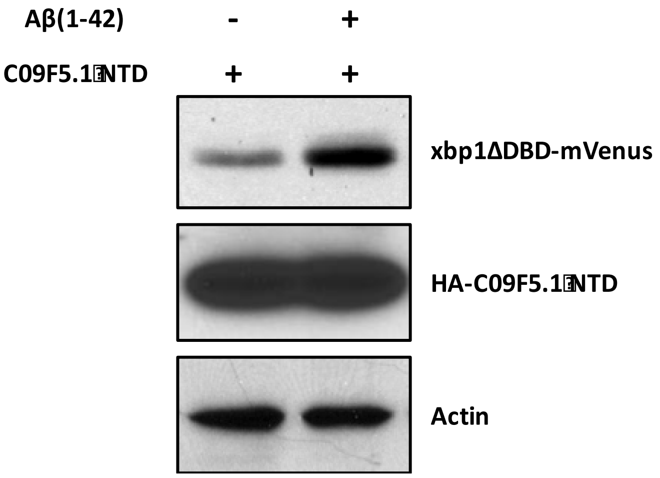

Figure S3. UPR activation by C09F5.1-NTD in ERAI-293T cells. XBP1ΔDBDmVenus was detected with anti-GFP antibody (A-11122, Invitrogen), and HA-C09F5.1-NTD was identified with anti-HA antibody (Y-11, Santa Cruz). β-actin was used as a loading control.

Table S1. Amino acid identity between C09F5.1 nematode homologues and ceC09F5.1.

	H. contortus **CDJ96801.1**			***T. canis*** **KHN74642.1**		
	N-terminal	C-terminal	BRICHOS	N-terminal	C-terminal	BRICHOS
Amino acid identity (%)	47	73	73	28	58	60
Query coverage (%)	97	96	100	97	96	100
	A. suum ERG82335.1			*B. malayi* XP_001900575.1		
	N-terminal	C-terminal	BRICHOS	N-terminal	C-terminal	BRICHOS
Amino acid identity (%)	29	56	59	36	53	55
Query coverage (%)	88	91	100	55	84	100

H. contortus-Haemonchus contortus, T. canis-Toxocara canis
A. suum-Ascaris suum, B. malayi-Brugia malayi

Table S2. Phosphorylation site prediction of C09F5.1 by c-Jun N-terminal kinases.

Position	Code	Kinase	Peptide	Score	Cutoff
83	S	CMGC/MAPK/JNK/JNK1	YGQHIETSPPPVQRY	3	1.892
83	S	CMGC/MAPK/JNK/JNK2	YGQHIETSPPPVQRY	30.794	6.118
83	S	CMGC/MAPK/JNK/JNK3	YGQHIETSPPPVQRY	10.625	6.781
83	S	CMGC/MAPK/JNK	YGQHIETSPPPVQRY	4.091	2.944
172	S	CMGC/MAPK/JNK	TRPSAMRSSSAAAQR	3.554	2.944
173	S	CMGC/MAPK/JNK	RPSAMRSSSAAAQRS	2.959	2.944
202	S	CMGC/MAPK/JNK/JNK3	SYRQEFASDNESLAR	6.812	6.781
280	T	CMGC/MAPK/JNK/JNK2	AFENSEYTPELLRSL	31.971	6.118
280	T	CMGC/MAPK/JNK/JNK3	AFENSEYTPELLRSL	10	6.781
280	T	CMGC/MAPK/JNK	AFENSEYTPELLRSL	3	2.944

Based on the Group-based Prediction System (GPS 3.0)
